# Surgical Decisions Based on a Balance between Malignancy Probability and Surgical Risk in Patients with Branch and Mixed-Type Intraductal Papillary Mucinous Neoplasm

**DOI:** 10.3390/jcm9092758

**Published:** 2020-08-26

**Authors:** Seung Jae Lee, Seo Young Park, Dae Wook Hwang, Jae Hoon Lee, Ki Byung Song, Woohyung Lee, Jaewoo Kwon, Yejong Park, Song Cheol Kim

**Affiliations:** 1 Department of Surgery, Konyang University Hospital, Daejeon 35365, Korea; leesj54gs@gmail.com; 2 Department of Clinical Epidemiology and Biostatistics, University of Ulsan College of Medicine and Asan Medical Center, Seoul 05505, Korea; biostat81@amc.seoul.kr; 3 Division of Hepatobiliary and Pancreatic Surgery, Department of Surgery, University of Ulsan College of Medicine and Asan Medical Center, Seoul 05505, Korea; dwhwang@amc.seoul.kr (D.W.H.); gooddr23@naver.com (J.H.L.); mtsong21c@amc.seoul.kr (K.B.S.); ywhnet@gmail.com (W.L.); skunlvup@naver.com (J.K.); blackpig856@gmail.com (Y.P.)

**Keywords:** intraductal papillary neoplasm of the pancreas, malignancy prediction, nomograms, ACS NSQIP surgical risk calculator, balance

## Abstract

Objective: To propose a decision tool considering both malignancy probability and surgical risk for intraductal papillary mucinous neoplasm (IPMN). Background Data Summary: Surgical risk and malignancy probability are both critical factors in making decisions about surgical resection of IPMN. Methods: We included 800 patients who underwent pancreatic resection for branch duct and mixed-type IPMN (April 1995 to June 2018). A nomogram was used to obtain the malignancy probability (MP-N). The surgical risks were estimated as the postoperative complication rate and serious complication from the ACS NSQIP surgical risk calculator (SC-ACS NSQIP). The risk–benefit analysis was conducted in two ways: calculation of the cutoff value of MP-N using the complication rate and directly comparing the MP-N and SC-ACS NSQIP results. Results: The optimal cutoff value of MP-N was 32% and 21% in the pancreaticoduodenectomy (PD) and distal pancreatectomy (DP) groups, respectively, when using the major complication rate (Clavien grades III over). When we applied the optimal cutoff value to the two surgical methods, surgery was reduced by 51.7% in the PD group and 56% in the DP group, and the AUC value of the malignant predictions were 0.7126 and 0.7615, respectively. According to the direct comparison of MP-N and SC-ACS NSQIP, surgery was reduced by 31.7%, and the AUC value of malignant prediction was 0.6588. Conclusion: Our risk–benefit analysis model considering both malignancy probability and surgical risk is relatively acceptable, and it may help surgeons and patients make treatment decisions for a disease with a broad spectrum of malignancy rates.

## 1. Introduction

Intraductal papillary mucinous neoplasms (IPMNs) are premalignant lesions of the pancreas [1]. IPMNs are also regarded as precursor of pancreas ductal adenocarcinoma (PDAC) and constitute an important system for studies of pancreatic tumorigenesis [2,3]. Accurate identification of IPMNs harboring either invasive carcinoma or high-grade dysplasia (HGD), which should undergo surgical resection, is the subject of extensive studies throughout the world. Currently, three guidelines provide recommendations on IPMN surgical resection and surveillance on the basis of malignancy probability: the American Gastroenterological Association (AGA) [4], the International Association of Pancreatology (IAP) [5,6], or the European Study Group on Cystic Tumours of the Pancreas (European) [7]. Both the 2017 IAP [5] and the 2018 European [7] guidelines recommend surgical resection for all surgically fit patients with the jaundice, the presence of an enhancing mural nodule ≥ 5 mm, the presence of a solid component, positive cytology for HGD or invasive cancer or a dilated pancreatic duct ≥10 mm. Compared with the 2017 IAP and 2018 European guidelines, the 2015 AGA [4] guideline is more conservative [8]. The 2015 AGA guideline recommend resection for main duct dilatation with the presence of a nodule or cytology positive for malignancy. Additionally, according to both the 2017 IAP and the 2018 European guidelines, acute pancreatitis caused by IPMN, an enhancing mural nodule <5 mm, a dilated pancreatic duct between 5 and 9.9 mm, or an increased level of serum carbohydrate antigen 19–9 without jaundice, are recommended for relative indication for surgical resection.

As the statistical significance of these various variables related to the prediction of malignancy is different, these guidelines recommendations are limited in predicting individual IPMN malignancy risk. In response to this limitation, a Korea–Japan collaborative study proposed the use of a nomogram predicting the individual risk of malignancy for a patient with IPMN and main duct dilatation of 10 mm or less [9]. This quantitative system would enable determination of individual risks, and it could be very useful in personalizing treatment of patients with BD-IPMN.

However, there is some difficulty in determining how the results of the nomogram can be applied to each patient because the cutoff value of a predicted malignancy probability from the nomogram to suggest surgery is unclear. In clinical practice, surgeons usually decide whether to perform surgery after considering the surgical risk, including the type of surgery, the patient’s age, and comorbidities. However, there no studies on surgical decision making that consider both the malignancy probability and the surgical risk in patients with IPMN.

The purpose of this study was to propose a surgical decision-making tool that considers the surgical risk as well as the malignancy probability in patients with IPMN. We retrospectively reviewed the preoperative variables, postoperative histologic findings, and postoperative complications in patients with IPMNs who underwent pancreatic resection. We also calculated the malignancy probability from the nomogram (MP-N) proposed in the Korea–Japan collaborative study [9] and the serious complications from the American College of Surgeons National Surgical Quality Improvement Program (ACS NSQIP) surgical risk calculator (SC-ASC NSQIP) [10].

## 2. Materials and Methods

### 2.1. Patients

All consecutive patients who underwent pancreatic resection for pathologically proven IPMN between April 1995 and June 2018 at a single institution were evaluated. Patients found to have a pathologically main duct (MD)–IPMN, main pancreatic duct (MPD) dilatation > 10 mm, or incomplete radiological or laboratory data for an accurate calculation of MP-N or SC-ACS NSQIP were excluded from further analysis. Finally, we included 800 patients. This study was approved by the Institutional Review Board of Asan medical center, and informed consent was waived due to the retrospective study design (IRB No. 2018–1314).

### 2.2. Radiologic and Endoscopic Imaging Analysis

All included patients underwent at least one or more preoperative abdominal imaging studies, including computed tomography (CT), magnetic resonance imaging (MRI) with magnetic resonance cholangiopancreatography (MRCP) and/or EUS within 4 weeks before surgery, either from an external institution with good image quality (except for EUS) or conducted at the study institution. We evaluated the following imaging parameters on CT, MRI, and EUS: MPD size, location of the cyst, cyst size, and presence and size of any mural nodule/solid component. The EUS findings were analyzed based on formal reports, and the CT and MRI images were retrospectively reviewed by two pancreatic surgeons with more than 15 years of experience and more than 5 years of experience, respectively. MPD size was measured at the maximum diameter of the MPD. Cyst location was categorized as head, body/tail, or diffuse. Cyst size was measured by the maximal cross-sectional diameter of the lesion. Mural nodules were defined as papillary excrescences or protuberances within the dilated BD or MPD [11,12].

### 2.3. Laboratory Values Analysis

Preoperative serum carbohydrate antigen (CA) 19–9 and carcinoembryonic antigen (CEA) levels were evaluated within 4 weeks before surgery. Reference values were defined as 37 U/mL and 5 ng/mL, respectively.

### 2.4. Pathological Classification

The diagnosis of IPMN was established following the current World Health Organization (WHO) guidelines for IPMN [13]. The IPMN type was categorized as MD-IPMN, BD-IPMN, or mixed IPMN. The histologic tumor grade was classified as low-grade dysplasia (LGD), intermediate-grade dysplasia (IGD), high-grade dysplasia (HGD), or invasive carcinoma. In this study, malignancy was defined as HGD or invasive IPMN.

### 2.5. Surgical Complication Analysis

Postoperative complications were classified according to the Clavien–Dindo classification [14] and the type of surgery. Major complications were defined as higher than grade III of the Clavien–Dindo classification.

### 2.6. Predicted Malignancy Probability from the Nomogram

The nomogram is freely available at http://statgen.snu.ac.kr/software/nomogramIPMN [9]. The factors included in the nomogram are age, sex, MPD diameter, cyst size, mural nodule, and serum tumor markers (CA19-9 and CEA). The MPD diameter and cyst size were the longest measured among the three imaging examinations (CT, MRI, and EUS). The presence of a mural nodule was confirmed in at least one of the three imaging examinations. The malignancy probability was then calculated with the nomogram.

### 2.7. Predicted Serious Complications from the ACS NSQIP Surgical Risk Calculator

The ACS NSQIP surgical risk calculator is freely available at https://riskcalculator.facs.org/RiskCalculator/index.jsp. This calculator estimates the risk of postoperative complications based on the type of surgery and the patient’s age, sex, body mass index (BMI), functional status, American Society of Anesthesiologists (ASA) classification, and comorbidities. The outcomes are presented as risk probabilities given in terms of percentages of various postoperative complications.

### 2.8. Risk–Benefit Analysis for Surgical Decisions

The benefits of surgery are in eliminating the risk of malignancy. The risk of malignancy was estimated using the MP-N. The risks of surgery were estimated in two ways, the postoperative complication rate of the study cohort given as a statistical risk, and the predicted SC-ACS NSQIP risk. The risk–benefit analysis was conducted in two ways: calculation of the cutoff value of MP-N using the complication rate, and directly comparing the MP-N and the SC-ACS NSQIP.

#### 2.8.1. Using the Complication Rate of the Study Cohort as a Statistical Risk

We defined the cutoff value of MP-N as the point at which the risk of missing malignant lesions and the risk of complications from unnecessary surgery were equal. The risk of missing malignant lesions was estimated as the number of pathologically confirmed malignant patients with an MP-N below the cutoff, defined as the number of false negatives (FN). The risk of complications from unnecessary surgery was estimated as the number of patients who developed surgical complications in pathologically confirmed benign patients with an MP-N above the cutoff as the number of false positives (FP) multiplied by the complication rate of the study cohort.

We could find the cutoff value of MP-N at which the FN, FP, and complication rate satisfied the following equation:

Number of false negatives (FN) = Number of false positives (FP) × complication rate(1)

We divided the subgroups according to the type of surgery since the surgical complication rates were different depending on the type of surgery. In addition, we applied four types of complication rates according to their severity. The cutoff value of MP-N was calculated according to each type of surgery and the severity of the complications.

#### 2.8.2. Using PSC-ACS NSQIP as Predicted Risk

The risk–benefit analysis using the predicted risk was conducted by directly comparing the MP-N and the SC-ACS NSQIP from each individual patient in the study cohort.

### 2.9. Statistical Analysis

Univariate and multivariable analyses were performed to identify predictive factors of malignancy. Continuous variables are summarized as mean ±standard deviation (SD) and were compared using Student’s t-test. Categorical variables are presented as count and percentage and were compared using the Chi-square test. Multivariable analyses of significant factors identified in the univariate analyses were performed using a logistic regression model. All tests were 2-sided, and p-values less than 0.05 were considered statistically significant.

The performances of the nomogram and the ACS NSQIP surgical risk calculator for our study population were quantified based on their discrimination and calibration. Discrimination was used to determine whether the individual predictions were correct. The level of discrimination was estimated using the area under the receiver operating characteristic (ROC) curve (AUC). Calibration was used to determine whether the observed true probabilities were concordant with the predicted probabilities. The level of calibration was tested using the Hosmer–Lemeshow test and described using a calibration plot.

The univariate and multivariable analyses were performed using SPSS version 22 (SPSS, Inc., Chicago, IL, USA), and the analyses of the ROC and the calibration were performed using R software version 3.5.1 (The R Foundation for Statistical Computing, Vienna, Austria).

## 3. Results

### 3.1. Study Population

**Figure 1:**
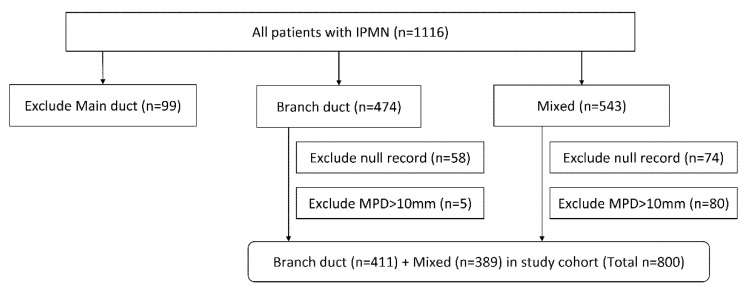
Flow diagram of the analysis by type of IPMN. IPMN, intraductal papillary mucinous neoplasm; MPD, main pancreatic duct.

Of the total 1116 patients who underwent pancreatic resection for histologically confirmed IPMN, including 99 MD-IPMN (8.9%), 474 BD-IPMN (42.5%), and 543 mixed IPMN (48.6%). Surgical pathology revealed that 435 patients had LGD (39.0%), 375 patients had IGD (33.6%), 165 patients had HGD (14.8%), and 141 patients had invasive lesions (12.6%). Malignant lesions were identified in 44 patients (44/99, 44.4%) with MD-IPMNs, 76 patients (76/474, 16.0%) with BD-IPMNs, and 184 patients (184/543, 27.2%) with mixed IPMNs. Exclusion criteria for further evaluation were any patients with MD-IPMN, at least 1 missing covariate, and MPD dilatation exceeding 10 mm on radiologic imaging or EUS. Then, the 800 patients who fit the criteria were included (Figure 1).

The preoperative and postoperative characteristics of the study population were analyzed. In univariate analysis, age, tumor location, MPD dilatation, cyst size, presence of a mural nodule, and serum CA19-9 were statistically significant predictors of malignancy. The predictive abilities of sex and serum CEA were not statistically significant (Supplement Table S1). In a multivariable logistic regression model that included the significant factors identified in the univariate analyses, MPD dilatation, the presence of a mural nodule, and elevated serum CA19-9 were statistically predictors of malignancy (Supplement Table S2). The postoperative complication rates according to their severity and the type of surgery are listed in Supplement Table S3. The Rate of POPF, DGE, PPH, chyle leak and 30-day mortality according to type of surgery are listed in Supplement Table S4.

### 3.2. Validation of the Nomogram and ACS NSQIP Surgical Risk Calculator

**Figure 2:**
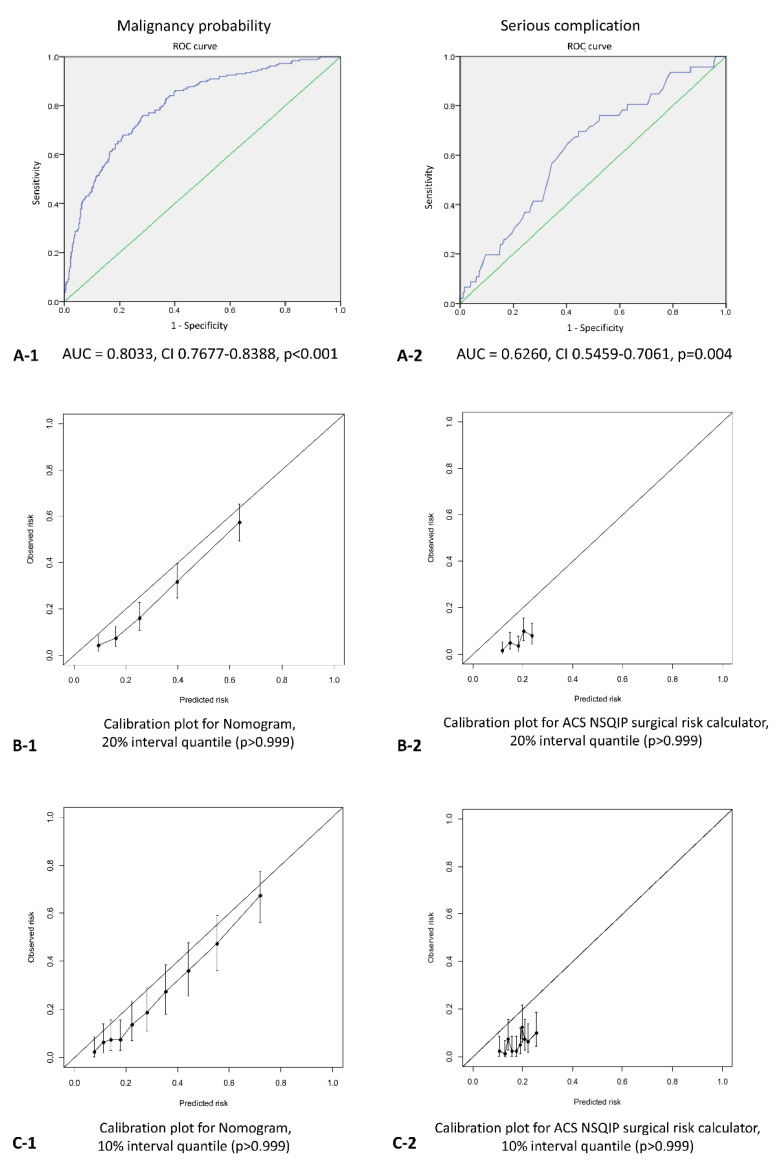
ROCs (**A**) and the Hosmer–Lemeshow tests in the 20% (**B**) and 10% (**C**) of the nomogram (1) and ACS NSQIP surgical risk calculator (2) in the study cohort.

The MP-N and the SC-ACS NSQIP were calculated for all 800 patients. The AUC for the MP-N was 0.8033 (95% CI 0.7677–0.8388, *p* < 0.001). The Hosmer–Lemeshow goodness-of-fit test showed the good calibration of the nomogram, both in 10% and 20% quantile scales (*p* > 0.999). The AUC for the SC-ACS NSQIP was 0.6260 (95% CI 0.5459–0.7061, *p* = 0.004). The Hosmer–Lemeshow goodness-of-fit test showed the poor fit of the surgical risk calculator, both in 10% and 20% quantile scales (*p* > 0.999) (Figure 2).

### 3.3. Risk–Benefit Analysis for Surgical Decision Making

#### 3.3.1. Using the Complication Rate of the Study Cohort as the Statistical Risk

The numbers of FNs and FPs at each possible cutoff were evaluated to determine the cutoff value of MP-N. The cutoff values, according to the severity of the complication and the type of surgery, were identified as described in the Methods section. The results are listed in Table 1 and Table 2. The optimal cutoff values according to the severity of the complications were 13, 21, 32, and 41% in the pancreaticoduodenectomy (PD) group, and 14, 16, 21, and 27% in the distal pancreatectomy (DP) group. The diagnostic characteristics of the malignancy predictions when each cutoff value was applied according to the severity of the complications are listed in Table 3 and Table 4. The AUC value was the highest when the cutoff value was determined using major complications in both the PD and DP groups (PD; 0.7126 and DP; 0.7615). After applying this cutoff value to the two groups, surgery was reduced by 51.7% and 56%, respectively.

#### 3.3.2. Using SC-ACS NSQIP as the Predicted Risk

Among the 800 patients, the MP-N was greater than the SC-ASC NSQIP in 546 patients. According to the risk–benefit analysis using the predicted risk, surgery was reduced by 31.7% in the study cohort. The sensitivity, specificity, positive predictive value, and negative predictive value of malignancy prediction were 92.6, 39.2, 31.9, and 94.5%, respectively. The AUC was 0.6588 (95% CI 0.6188–0.6989, *p* < 0.001) (Table 5). Similar results were obtained after exclusion of small subgroups that underwent total or central pancreatectomy, or enucleation (Supplement Table S5).

## 4. Discussion

In the present study, a nomogram from a Korea–Japan collaborative study [9] was used to predict the probability of malignancy in individual patients with IPMNs. This nomogram was based on a large eastern cohort, includes both radiologic and laboratory findings, and it has the advantage of easy access. In addition, studies have recently been published, showing that it also has good external validity for western cohorts [15]. The current study demonstrated the MP-N to have good calibration with the Hosmer–Lemeshow goodness-of-fit test, and that it had a good AUC value of 0.8033 in patients with IPMN and main duct dilatation of 10 mm or less. The authors who originally proposed the nomogram suggested a cutoff value of 30 to 40% for predicting the probability of malignancy; however, there seems to be no specific rationale or further studies supporting this recommendation. Thus, the current study conducted a risk–benefit analysis considering both malignancy probability and surgical risk to find the optimal cutoff value of MP-N that is applicable in clinical practice and is evidence-based. The risk–benefit analysis was conducted in two ways: using the cohort complication rate as the statistical risk, and the calculated individual surgical risk as the predicted risk.

The complication rate and the cutoff values were both estimated by dividing the group according to the surgical procedure. The patients who underwent total or central pancreatectomy, or enucleation are too small for separate analysis, so they were excluded in risk benefit analysis using statistical risk. In general, the PD procedure has a higher complication rate than the DP procedure, and the same results were obtained in our current study [16,17,18,19]. A higher cutoff value was selected for the PD group than the DP group because of the higher complication rate. This means that even if the lesions are practically identical, different decisions about treatment are possible if their locations are different. In practice, pancreatic surgeons may use different criteria based on the location of the pancreatic IPMN to determine the type of surgery. Our study results suggest that dividing groups according to the surgical procedure and presenting different optimal cutoff values may be helpful in surgical decision making in clinical practice.

The cutoff value will vary depending on which complication rate is applied. We used four different complication rates based on their severity. The severity of the surgical complications to be compared with the risk of malignancy will vary by surgeon. Depending on the surgeon’s judgment, the criteria could be applied differently. In the present study, when the major complication rate was used, we confirmed that the AUC for malignancy prediction was the highest in both the PD and DP groups. However, the cutoff values recommended in our cohort are not absolute. Each institution or surgeon can set different optimal cutoff values based on their pancreatic surgery results.

The hospital and surgical volume is known to be associated with the risk of complication in pancreatic surgery [20,21]. The institution that conducted present study is a high-volume center performing more than 500 cases of pancreatic surgery per year and there may be differences in the rate of complication from low volume center. Other high-volume centers are expected to be similar to this study and may be referred to this study results, however, surgical decisions should be made based on the rate of complications in each institution. The surgical risks were estimated from ACS NSQIP surgical risk calculator to further generalize the differences in the complication rate of the institutions.

AUC value of SC-ACS NSQIP was relatively low (0.6260), and the Hosmer–Lemeshow goodness-of-fit test showed it was poorly calibrated. There are several possible explanations for this. First, the ACS NSQIP surgical risk calculator was developed from US hospital data [10]. Our study cohort included only Koreans, and the ACS NSQIP surgical calculator for pancreatic surgery has never been validated in an eastern population. Second, the ACS NSQIP surgical calculator includes only 30-day postoperative outcomes. However, our study included both immediate complications and late complications, defined as those occurring 30 days postoperatively. The inclusion of late complications can more accurately assess the risk of surgery. Especially in PD, late complications such as anastomotic strictures or marginal ulcers often occur. In our study cohort, serious late complications were estimated to comprise 39.0% of overall major complications. Further studies are needed for the future development of pancreatectomy specific risk models that include late complications.

The comparison of the surgical risk and the malignancy probability of each individual patient is more appropriate for deciding on patient-specific treatment than the determination of the cutoff value using complication rates in a study population. We directly compared the MP-N and SC-ACS NSQIP. Because the risk of surgery becomes the cutoff value, it can be a problem if the risk of surgery is too low. However, in the case of pancreatic resections applied to the ACS NSQIP surgical risk calculator, we have a proper minimal serious complication value (PD; 12.2% and DP; 8.4%). Despite its low validity, when the risk of surgery is determined individually using the ACS NSQIP surgical calculator, it is relatively acceptable in combination with the malignancy risk for making treatment decisions. If the surgical decision was made based on our risk–benefit analysis model, the ACS NSQIP surgical risk calculator may be helpful for institutions with limited pancreatic surgical data.

Recently, several nomograms to predict malignancy in IPMN have been proposed to overcome the limitation of current guidelines [9,22,23,24]. However, in actual clinical practice, management of IPMNs is still being performed according to the current guidelines from the AGA, IAP, or European, because most nomograms lack external validation. A recent systematic review by van Huijgevoort et al. [8] summarized the current guidelines of surgical indications. When applying these indications for surgical resection into the nomogram, the patients with fit the surgical indications such as the presence of mural nodule, the MPD dilatation, or the elevated CA19-9, are calculated to be mostly more than the proposed optimal cutoff value (PD; 32% and DP; 21%).

IPMN lesions have a chance of undergoing a malignant transformation during the patient’s lifetime. Han Y et al. [25] reported the median annual growth rate of a cyst was 0.8 mm over a median follow-up time of 61 months in patients with BD-IPMN. Early on, the malignant potential of this entity led to surgical resection of most pancreatic IPMNs. The present study also included many patients with relatively low malignant risk who underwent pancreatectomy before the current guidelines were published. However, according to the recently published guidelines, the tendency to perform serial observations has increased. Deciding upon follow-up observations instead of surgery is a possibility, but the downsides of lifelong surveillance can involve the risk of missing a malignancy, the inconvenience of regular hospital visits, and the high costs of the examinations. If the risk of surgery is not high, surgery will not only eliminate a potential malignancy and the possibility of the progression of the lesions over time, but will also reduce medical costs over the long term. The results of the present study provide new perspectives to patients with IPMN, allowing them the opportunity to choose other treatment options. The risk of surgery, of course, is a necessary means of making surgical decisions in patients with IPMN. The patients need to be discussed by a multidisciplinary team with experts before surgical decision-making.

The present study has several limitations. First, measurement bias may have occurred because imaging findings that have inter-observer differences were included in the calculation of malignancy probability. Second, the validity verification of malignancy probability from nomogram may be skewed, as 47% of those in this study are already included in the multicenter study that had already proposed nomogram [9]. Third, the ACS NSQIP surgical risk calculator showed poor validity in our study cohort. The risk benefit analysis using ACS NSQIP surgical risk calculator is limited to clinical applications for patients with low validity. If more accurate surgical risk predictions are available, we may be able to establish a more tailored treatment plan for the individual patient with IPMN. The authors are planning to develop a surgical decision-making model using a clinical decision support system, which uses a form of artificial intelligence called machine learning. Forth, this was a retrospective single-center study and only included surgical patients. Although the malignancy rates in our study were comparable with those of previous studies [26,27,28,29,30], our risk–benefit analysis requires external validation in other surgical patients. Prospective studies using our risk–benefit analysis model in newly diagnosed patients or in patients undergoing surveillance for IPMN are also needed. Finally, we missed discussing the biological factors about the IPMN and early pancreatic tumorigenesis. KRAS and GNAS mutations contribute to progression from IPMN to PDAC [2,3]. TGF-β signaling is play a different role depending on the timing of the pancreatic tumorigenesis, potentially affecting surgical success [31,32]. Evaluation of these biological landscapes may give more information for the surgical resection and surveillance of the patients with IPMNs.

## 5. Conclusions

Our risk–benefit analysis model considering both malignancy probability and surgical risk is relatively acceptable for predicting malignancy and for the reduction of major complications in patients with high surgical risk. It may help surgeons and patients to make treatment decisions for a disease with a broad spectrum of malignancy rates. Each institution can set different cutoff values based on their surgical complication rates. The ACS NSQIP surgical risk calculator may be helpful for institutions with limited pancreatic surgical data. Further studies are needed for the future development of specific malignant probability and surgical risk balance models to determine a more tailored treatment plan for each individual IPMN patient.

## Figures and Tables

**Table 1:** Variables of calculation method according to the cutoff and the severity of complications in the pancreaticoduodenectomy group (*n* = 464).

Cutoff (%)	FP	FN	FP × Complication Rate (%)			
			≥Grade II (43.3)	≥Grade IIIa (18.1)	≥Grade IIIb (6.7)	≥Grade IV (2.4)
**10**	**319**	2	138.127	57.739	21.373	7.656
**13**	288	**6**	124.704	52.128	19.296	**6.912**
14	274	8	118.642	49.594	18.358	6.576
15	266	8	115.178	48.146	17.822	6.384
20	208	13	90.064	37.648	13.936	4.992
**21**	203	**14**	87.899	36.743	**13.601**	4.872
22	196	15	84.868	35.476	13.132	4.704
30	148	22	64.084	26.788	9.916	3.552
**32**	131	**23**	56.723	**23.711**	8.777	3.144
33	125	26	54.125	22.625	8.375	3
34	117	29	50.661	21.177	7.839	2.736
40	89	35	38.537	16.109	5.963	2.136
**41**	86	**37**	**37.238**	15.566	5.762	2.064
42	82	38	35.506	14.842	5.494	1.968

Shown in bold with underline is the cutoff value for each complication rate satisfying the following equation: Number of false negatives = Number of false positives × complication rate. FP indicates false positive; FN, false negative.

**Table 2:** Variables of calculation method according to the cutoff and the severity of complications in the distal pancreatectomy group (*n* = 293).

Cutoff (%)	FP	FN	FP × Complication Rate (%)			
			≥Grade II (30.4)	≥Grade IIIa (8.5)	≥Grade IIIb (3.4)	≥Grade IV (2.0)
**10**	**188**	0	57.152	15.98	6.392	3.76
13	149	2	45.296	12.665	5.066	2.98
**14**	138	**3**	41.952	11.73	4.692	**2.76**
15	127	4	38.608	10.795	4.318	2.54
**16**	118	**4**	35.872	10.03	**4.012**	2.36
17	107	4	32.528	9.095	3.638	2.14
18	103	5	31.312	8.755	3.502	2.06
20	86	6	26.144	7.31	2.924	1.72
**21**	82	**7**	24.928	**6.97**	2.788	1.64
22	78	8	23.712	6.63	2.652	1.56
25	59	11	17.936	5.015	2.006	1.18
26	56	13	17.024	4.76	1.904	1.12
**27**	54	**16**	**16.416**	4.59	1.836	1.08
30	46	20	13.984	3.91	1.564	0.92

Shown in bold with underline is the cutoff value for each complication rate satisfying the following equation: Number of false negatives = Number of false positives × complication rate. FP indicates false positive; FN, false negative.

**Table 3:** Diagnostic characteristics of malignancy prediction according to the cutoff and the severity of complications in the pancreaticoduodenectomy group (*n* = 464).

Complication Rate	Cutoff Value	Number under Cutoff (%)	Sensitivity	Specificity	PPV	NPV	AUC
≥grade IV	13%	66 (14.2)	0.9483	0.1724	0.2764	0.9091	0.5158
≥grade IIIb	21%	159 (34.3)	0.8793	0.4167	0.3344	0.9119	0.6480
≥grade IIIa	32%	240 (51.7)	0.8017	0.6236	0.4152	0.9042	0.7126
≥grade II	41%	299 (64.4)	0.6810	0.7529	0.4788	0.8762	0.4986

AUC indicates area under the receiver operating characteristic curve; NPV, negative predictive value; PPV, positive predictive value.

**Table 4:** Diagnostic characteristics of malignancy prediction according to the cutoff and the severity of complications in the distal pancreatectomy group (*n* = 293).

Complication Rate	Cutoff Value	Number under Cutoff (%)	Sensitivity	Specificity	PPV	NPV	AUC
≥grade IV	14%	104 (35.5)	0.9444	0.4226	0.2698	0.9712	0.6835
≥grade IIIb	16%	125 (42.7)	0.9259	0.5523	0.2976	0.9680	0.7161
≥grade IIIa	21%	164 (56.0)	0.8704	0.6527	0.3615	0.9571	0.7615
≥grade II	27%	201 (68.6)	0.7037	0.7741	0.4130	0.9204	0.5346

AUC indicates area under the receiver operating characteristic curve; NPV, negative predictive value; PPV, positive predictive value.

**Table 5:** Diagnostic characteristics of malignancy prediction according to the risk–benefit analysis using the predicted risk in the study cohort (*n* = 800).

Cutoff	Number above the Cutoff (%)	Sensitivity	Specificity	PPV	NPV	AUC
SC-ACS NSQIP	546 (68.3)	0.9255	0.3922	0.3187	0.9449	0.6588

AUC indicates area under the receiver operating characteristic curve; NPV, negative predictive value; PPV, positive predictive value; PSC-ACS NSQIP, predicted serious complications from the American College of Surgeons National Surgical Quality Improvement Program surgical risk calculator.

## Supplementary Materials

The following are available online at https://www.mdpi.com/2077-0383/9/9/2758/s1. Table S1: Demographic and clinical features predicting malignancy in the study cohort; Table S2: Multivariable analysis for demographic and clinical features predicting malignancy in the study cohort; Table S3: Complication rate according to the severity and the type of surgery in the study cohort (*n* = 800); Table S4. Rate of POPF, DGE, PPH, chyle leak and 30-day mortality according to type of surgery in the study cohort (*n* = 800); Table S5: Diagnostic characteristics of malignancy prediction according to the risk-benefit analysis using the predicted risk in PD and DP group (*n* = 757).
