# Supplementary material for: Surgical Decisions Based on a Balance between Malignancy Probability and Surgical Risk in Patients with Branch and Mixed-Type Intraductal Papillary Mucinous Neoplasm
Source: J Clin Med. 2020 Aug 26;9(9):2758. doi: 10.3390/jcm9092758 (PMC7565903; doi:10.3390/jcm9092758)
Supplement: Supplementary file 1 [file jcm-09-02758-s001.pdf]

Supplement Table 1. Demographic and clinical features predicting malignancy in the study cohort.

Features	ALL (n = 800)	Malignancy (n = 188)	Benign (n = 612)	P-value
Age (year)	62.4 ± 9.1	63.6 ± 10.1	62.0 ± 8.8	0.043
Sex				0.063
Male	490 (61.3%)	126 (70.1%)	364 (53.8%)	
Female	310 (38.7%)	62 (29.9%)	248 (46.2%)	
Location				0.019
Head	467 (58.4%)	121 (64.4%)	346 (56.5%)	
Body / Tail	315 (39.4%)	59 (31.4%)	256 (41.8%)	
Diffuse	18 (2.2%)	8 (4.2%)	10 (1.7%)	
Main duct diameter (mm)	4.1 ± 2.7	5.7 ± 2.8	3.6 ± 2.4	<0.001
Cyst size (mm)	32.1 ± 13.6	35.2 ± 15.4	31.2 ± 12.8	<0.001
Mural nodule (+)	317 (39.6%)	136 (72.3%)	181 (29.6%)	<0.001
Serum CEA (ng/mL)	2.6 ± 4.4	3.0±6.6	2.4±3.5	0.206
Serum CA19-9 (U/mL)	23.1 ± 126.3	55.6 ± 256.4	13.1 ± 17.8	<0.001

Supplement Table 2. Multivariable analysis for demographic and clinical features predicting malignancy in the study cohort.

Variable	Multivariate analysis	
	OR (95% CI)	P-value
Age	1.000 (0.979–1.021)	0.966
Location (head vs. body / tail)	1.333 (0.874–2.034)	0.182
Main duct diameter	1.274 (1.185–1.370)	<0.001
Cyst size	1.013 (0.998–1.028)	0.087
Mural nodule (positive vs. negative)	5.720 (3.783–8.650)	<0.001
Serum CA19-9	1.015 (1.008–1.022)	<0.001

Supplement Table 3. Complication rate according to the severity and the type of surgery in the study cohort (*n* = 800).

Type of surgery	Complication rate				
	Total (%)	≥grade II (%)	≥grade IIIa (%)	≥grade IIIb (%)	≥grade IV (%)
PD/PPPD/SSPPD (n = 464)	232 (50.0)	201 (43.3)	84 (18.1)	31 (6.7)	11 (2.4)
DP (n = 293)	96 (32.8)	89 (30.4)	25 (8.5)	10 (3.4)	6 (2.0)
TP (n = 20)	8 (40.0)	6 (30.0)	3 (15.0)	3 (15.0)	0 (0.0)
CP with PJ (n = 13)	8 (61.5)	8 (61.5)	3 (23.1)	2 (15.4)	1 (7.7)
Enucleation (n = 10)	4 (40.0)	3 (30.0)	3 (30.0)	0 (0.0)	0 (0.0)
Total (n = 800)	349 (43.6)	307 (38.4)	118 (14.8)	46 (5.8)	18 (2.3)

PD indicates pancreaticoduodenectomy; PPPD, pylorus preserving PD; SSPPD, subtotal stomach preserving PD; DP, distal pancreatectomy; TP, total pancreatectomy; CP, central pancreatectomy, PJ, pancreaticojejunostomy.

Supplement Table 4. Rate of POPF, DGE, PPH, chyle leak and 30-day mortality according to type of surgery in the study cohort (*n* = 800).

Type of complication	Type of surgery					
	PD/PPPD/SSPPD (%), n = 464	DP (%), n = 293	TP (%), n = 20	CP with PJ (%), n = 13	Enucleation (%), n = 10	Total (%), n=800
POPF	73 (15.7)	29 (9.9)	-	3 (23.1)	2 (20)	107 (13.4)
Biochemical leak	53 (11.4)	18 (6.1)	-	3 (23.1)	-	74 (9.3)
Grade B	20 (4.3)	11 (3.8)	-	-	2 (20)	33 (4.1)
Grade C	-	-	-	-	-	-
DGE	13 (2.8)	1 (0.3)	0 (0)	0 (0)	0 (0)	14 (1.8)
Grade A	9 (2.0)	1 (0.3)	-	-	-	10 (1.3)
Grade B	2 (0.4)	-	-	-	-	2 (0.2)
Grade C	2 (0.4)	-	-	-	-	2 (0.2)
PPH	13 (2.8)	7 (2.4)	0 (0)	1 (7.7)	0 (0)	21 (2.6)
Grade A	1 (0.2)	1 (0.3)	-	-	-	2 (0.2)
Grade B	6 (1.3)	2 (0.7)	-	-	-	8 (1)
Grade C	6 (1.3)	4 (1.4)	-	1 (7.7)	-	11 (1.4)
Chyle leak	30 (6.5)	4 (1.4)	2 (10)	0 (0)	1 (10)	37 (4.6)
Grade A	22 (4.8)	3 (1.0)	-	-	1 (10)	26 (3.2)
Grade B	8 (1.7)	1 (0.4)	2 (10)	-	-	11 (1.4)
Grade C	-	-		-	-	-
30-day mortality	1 (0.2)	0 (0)	0 (0)	0 (0)	0 (0)	1 (0.1)

POPF indicates postoperative pancreatic fistula; DGE, delayed gastric emptying; PPH, postpancreatectomy hemorrhage; PD, pancreaticoduodenectomy; PPPD, pylorus preserving PD; SSPPD, subtotal stomach preserving PD; DP, distal pancreatectomy; TP, total pancreatectomy; CP, central pancreatectomy, PJ, pancreaticojejunostomy.

Supplement Table 5. Diagnostic characteristics of malignancy prediction according to the risk-benefit analysis using the predicted risk in PD and DP group (*n* = 757).

Cutoff	Number above the cutoff (%)	Sensitivity	Specificity	PPV	NPV	AUC
SC-ACS NSQIP	515 (68.0)	0.9176	0.3884	0.3029	0.9421	0.6530

PD indicates pancreaticoduodenectomy; DP, distal pancreatectomy; AUC, area under the receiver operating characteristic curve; NPV, negative predictive value; PPV, positive predictive value; PSC-ACS NSQIP, predicted serious complications from the American College of Surgeons National Surgical Quality Improvement Program surgical risk calculator.
